# Choroidal vascular index in patients with open angle glaucoma and preperimetric glaucoma

**DOI:** 10.1371/journal.pone.0213336

**Published:** 2019-03-20

**Authors:** Yuli Park, Kyong Jin Cho

**Affiliations:** Department of Ophthalmology, Dankook University Hospital, Dankook University College of Medicine, Cheonan, Korea; University of California San Diego, UNITED STATES

## Abstract

**Purpose:**

To evaluate choroidal structural changes in glaucoma using choroidal vascularity index (CVI) compared to healthy subjects.

**Methods:**

This retrospective study included 56 patients with open angle glaucoma (OAG), 50 patients with preperimetric glaucoma (PPG) and 50 age-matched healthy eyes. Choroidal images were binarized into luminal area (LA) and stromal area. CVI was defined as the ratio of LA to total circumscribed choroid area (TCA). Mean choroidal thickness (CT) and mean CVI between glaucoma patients and healthy subjects were compared.

**Results:**

OAG and PPG eyes showed smaller LA (0.45 ± 0.13 ㎟ vs. 0.47 ± 0.11 ㎟, *p* = 0.04). In multivariate regression analysis, CVI of both OAG (64.34±0.19%, *p* = 0.001) and PPG (65.37±0.15%, *p* = 0.001) were significantly lower than healthy eyes (68.81±0.14%).

**Conclusion:**

Eyes with glaucoma demonstrated reduced CVI compared with healthy eyes. CVI may be a potential noninvasive tool for studying vascular dysfunction in glaucoma.

## Introduction

Glaucoma is a progressive optic neuropathy that can cause irreversible blindness [1]. Early diagnosis aims to slow disease progression to prevent functional vision loss. Although the exact mechanism of glaucoma is unknown, increased intraocular pressure (IOP) is the main risk factor for glaucoma [2]. Considering that glaucomatous optic neuropathy can develop and progress in untreated eyes with statistically normal IOP, or in eyes with an IOP lowered by medical or surgical treatment, the pathogenesis of glaucoma progression is not solely dependent on IOP.

Vascular factors have been thought to be associated in the development and progression of the glaucoma [3–5]. In patients with glaucoma, blood flow of the choroid, retina, and the optic nerve head (ONH) have been measured to be reduced as compared to healthy normal eyes [6]. The role of the choroid in the pathogenesis of glaucoma is valuable to evaluate as choroidal circulation supplies some regions of the ONH. An abnormal blood supply to the peripapillary choroid has also been hypothesized to affect the pathogenesis of glaucoma [7]. Since the choroid is composed of connective tissue, melanocytes, blood vessels and nerves, measurement of choroidal thickness (CT) does not specify which choroidal components may be damaged. This limits the use of CT because both stromal and vascular components contribute to thickness.

Recently, swept-source OCT (SS-OCT) uses a long wavelength light source that allows for the identification of deep ocular structure. It incorporates a light source at 1050 nm wavelength and acquires 100,000 A-scans per second. SS-OCT, compared with spectral domain OCT, offers greater sensitivity at greater scanning depths [8].

Choroidal vascularity index (CVI) has been presented as a new tool to assess choroidal vascular status [9]. Sonoda *et al*. described a method for computing luminal and interstitial areas in the choroid to quantify choroidal vascular status [10]. However, to the best of our knowledge, no studies have been conducted to compare CVI specifically in open angle glaucoma (OAG) and preperimetric glaucoma (PPG) patients to healthy subjects. In this study, we aimed to determine the difference in the proportion of choroidal vasculature in patients with glaucoma and in healthy controls.

## Materials and methods

This retrospective, comparative study evaluated patients with OAG, PPG, and normal eyes from October 2015 to September 2018. Our study adhered to the tenets of the Declaration of Helsinki for research involving human subjects and followed all guidelines for investigation in human subjects required and approved by the Institutional Review Board of Yeouido St. Mary’s Hospital (IRB#SC12RISI0037). Written Consent was not obtained since this is retrospective study. Participating in the study were 56 eyes affected by OAG, 50 eyes of PPG, and 50 eyes of age-matched normal eyes.

Each patient underwent a comprehensive ophthalmologic evaluation including slit-lamp biomicroscopy, best-corrected visual acuity (BCVA), gonioscopy, Goldmann applanation tonometry, dilated stereoscopic examination of the ONH, color disc photography, standard automated perimetry (SAP) using the 24–2 Swedish Interactive Threshold Algorithm standard program (Humphrey Visual Field Analyzer; Carl Zeiss-Meditec Inc., Dublin, CA, USA), and SS OCT (DRI-OCT system; Topcon, Tokyo, Japan). Central corneal thickness (CCT) and axial length (AL) were measured using ultrasound pachymetry (Tomey, Nagoya, Japan) and IOL Master (Carl Zeiss Meditec Inc., Dublin, CA, USA).

All included eyes required BCVA of >20/40 and we included only subjects with open angles on gonioscopy. Subjects were excluded if they had history of ocular trauma or ocular surgery, retinal diseases, media opacity that could affect the quality of photography, optic nerve disease other than glaucoma, history of cerebrovascular event, or systemic medication use that could affect the visual field (VF), or systemic disease such as diabetes mellitus or hypertension. Inclusion criteria for eyes with OAG included (1) history of pretreatment IOP >21mmHg, (2) glaucomatous optic disc (diffuse or focal thinning of the neuroretinal rim), and (3) repeatable (≥2 consecutive) presence of VF defects consistent with glaucoma. Inclusion criteria for age-matched normal eyes included (1) an IOP <21 mmHg with no history of elevated IOP, (2) an absence of glaucomatous optic disc appearance, and (3) normal VF testing results. PPG patients met the same criteria for ONH and retinal nerve fiber layer (RNFL) changes as did the OAG (disc hemorrhage, focal thinning of the RNFL, typical neuroretinal rim loss or notching, or vertical elongation of the optic cup), but they did not show loss of VF. Eyes with consistently unreliable visual fields were also excluded.

SS OCT was performed in all patients. Two scans, including 1 macular scan centered on the fovea (7 x 7 mm protocol) and 1 peripapillary scan centered on the optic disc (6 × 6 mm protocol), were acquired. The ganglion cell–inner plexiform layer (GCIPL) between the outer boundary of the RNFL and the outer boundary of the inner plexiform layer (IPL) was measured at the macular region using a specific automatic segmentation algorithm. The average and 6 sectorial GCIPL thicknesses were measured from the circular annulus centered on the fovea. Bruch’s membrane and the chorioscleral interface were delineated with the machine’s built-in autosegmentation software and the subfoveal choroidal thickness (SFCT) was automatically measured by built-in calliper. The optic disc center was automatically detected, and a 3.4 mm diameter circle was drawn around it. With the built-in analysis software, the RNFL boundary was automatically segmented and the RNFL thickness throughout the scan was calculated. A 26 x 26 cube-grid centered in the optic disc was generated to automatically measure peripapillary CT (PCT). This grid included cubes around the optic nerve head and the delimitation of the optic nerve and the central cubes is automatically performed by the OCT software. Only high-quality scans, those with a TopQ Image Quality ≥ 60 and without involuntary saccade, overt misalignment, decenteration or blinking artifacts, were used for the analysis. The OCT scans were binarized using public domain software, Image J. Bruch’s membrane was the upper border and choroidal-scleral interface was the lower border and those were marked. The subfoveal choroidal area with width of 1500 μm (750 μm either side of the fovea) was marked. In the binarized images, dark pixels corresponded to the lumen of blood vessels and white pixels to the stroma [10]. The image was converted into red, green, and blue colors to allow the color threshold tool to select the dark pixels [9,11]. The stromal area (SA) and was calculated by subtracting LA from the total choroidal area (TCA). CVI was defined as the ratio of LA to TCA. The images with inconspicuous choroid scleral junction or motion artifacts were excluded from the image binarization and further analysis.

### Statistical analysis

Mann–Whitney U test was used to compare the means between different subjects. Univariate regression analyses were performed to determine whether demographics or ocular factors could influence CT and CVI. Multivariate linear regression analysis of SFCT and CVI was performed to assess the differences between glaucomatous and healthy eyes after adjusting for age and AL. All of statistical analyses were performed using SPSS version 20.0 (SPSS, Inc., Chicago, IL, USA) and *p* value of <0.05 was considered to be statistically significant.

## Results

Our study evaluated 56 eyes affected by OAG, 50 eyes of PPG, and 50 eyes of age-matched normal eyes. The mean age of the OAG, PPG, and healthy subjects were 69.4 ± 9.3, 64.9 ± 8.7, 67.3 ± 9.1 years respectively. There was not a statistically significant difference between groups in age, gender, BCVA, IOP, and CCT (*p* > 0.05). The mean number of glaucoma medications used during the study was 2 eye drops in OAG group and none used in PPG and healthy eyes. The demographic and clinical characteristics of subjects, stratified by diagnostic category, are summarized in Table 1.

**Table 1:** Comparison of demographic and ocular characteristics of glaucomatous patients and healthy subjects.

	OAG (n = 56)	PPG (n = 50)	Normal (n = 50)
Age (yr)	69.4 ± 9.3	64.9 ± 8.7	67.3 ± 9.1
Gender, male	29	18	25
AL (mm)	23.6 ± 2.8	24.2 ± 2.1	23.8 ± 1.9
CCT (㎛)	542.7 ± 29.5	553.3 ± 32.9	549.5 ± 30.4
BCVA (LogMAR)	0.16 ± 0.15	0.15 ± 0.13	0.13 ± 0.12
IOP (mmHg)	19.2 ± 3.4	15.7 ± 2.9	13.5 ± 2.1
Visual field MD (dB)	-5.4 ± 2.1	0.17 ± 0.94	0.01 ± 0.43
PSD (dB)	7.4 ± 3.7	1.8 ± 0.6	0.9 ± 0.5
RNFLT (㎛)	80.6 ± 15.4	94.8 ± 14.5	113.4 ± 9.6
GCIPLT (㎛)	101.0 ± 12.9	109.4 ± 10.8	119.7 ± 8.7

Mean value ± SD with 95% confidence intervals presented for normally distributed variables. AL = axial length; CCT = central corneal thickness; GCIPLT = ganglion cell-inner plexiform layer thickness; IOP = intraocular pressure; MD = mean deviation; PSD = pattern standard deviation; RNFLT = retinal nerve fiber layer thickness.

Univariate analysis of demographic and ocular factors showed that age and AL were significantly correlated with SFCT and PCT, however, not with CVI (Table 2).

**Table 2:** Univariate regression analysis of factors associated with peripapillary choroidal thickness (PCT), subfoveal choroidal thickness (SFCT), and choroidal vascularity index (CVI) for glaucomatous and healthy eyes.

		β coefficient	P value
SFCT	Age (years)	-4.37	0.001
	Gender (male)	0.52	0.31
	IOP (mmHg)	0.19	0.54
	Axial length (mm)	-15.74	0.02
	BCVA (LogMAR)	0.36	0.27
PCT	Age (years)	-8.95	0.002
	Gender (male)	1.72	0.46
	IOP (mmHg)	-2.05	0.38
	Axial length (mm)	-13.84	0.01
	BCVA (LogMAR)	0.04	0.69
CVI	Age (years)	-0.12	0.07
	Gender (male)	0.48	0.63
	IOP (mmHg)	-1.73	0.49
	Axial length (mm)	-2.76	0.13
	BCVA (LogMAR)	0.15	0.38

In Table 3, we compared healthy eyes with OAG and PPG eyes. OAG and PPG eyes had lower mean SFCT than healthy eyes (171.57 ± 84.24㎛, 174. 63 ± 87.69㎛, and 201.47 ± 91.03㎛, respectively) and also lower PCT than healthy eyes (115.43 ± 45.74㎛, 119.38 ± 49.26㎛, and 155.04 ± 48.95㎛, respectively) but those did not show statistically significant differences. OAG and PPG eyes had significantly lower LA than healthy eyes (0.45±0.13㎟, 0.47±0.11㎟, and 0.55±0.14㎟, respectively). There was no statistically significant difference in SA and TCA. The CVI was 64.34±0.19% for OAG eyes, 65.37±0.15% for PPG eyes, and 68.81±0.14% for healthy eyes. The CVI for both OAG and PPG eyes were significantly lower than healthy eyes (*p* = 0.001, both). The mean difference of CVI between OAG and PPG eyes was not statistically significant.

**Table 3:** Comparison of ocular characteristics, peripapillary choroidal thickness (PCT), subfoveal choroidal thickness (SFCT), and choroidal vascular index (CVI) between glaucomatous and normal eyes.

	OAG	PPG	Normal	*P* value (OAG vs. Normal)	*P* value (PPG vs. Normal)	*P* value (OAG vs. PPG)
SFCT (㎛)	171.57±84.24	174.63±87.69	201.47±91.03	0.07	0.09	0.27
PCT (㎛)	115.43±45.74	119.38±49.26	155.04±48.95	0.11	0.26	0.61
LA (㎟)	0.45±0.13	0.47±0.11	0.55±0.14	0.02	0.04	0.14
SA (㎟)	0.24±0.09	0.25±0.09	0.26±0.10	0.35	0.27	0.75
TCA (㎟)	0.70±0.22	0.72±0.20	0.81±0.24	0.14	0.58	0.39
CVI (%)	64.34±0.19	65.37±0.15	68.81±0.14	0.001	0.001	0.08

Multivariate linear regression analyses for the mean SFCT and CVI for OAG, PPG, and healthy eyes are presented in Table 4. After adjusting for age, AL, and SFCT, CVI of OAG and PPG were significantly reduced when compared to healthy eyes.

**Table 4:** Multivariate linear regression analyses for the choroidal vascularity index (CVI) and mean subfoveal choroidal thickness (SFCT) in OAG, PPG, and healthy eyes.

	**Unadjusted**			**Adjusted for age and axial length**	
	**Mean SFCT (㎛)**	**Mean differences (㎛)**	**P values**	**Mean differences (㎛)**	**P values**
OAG	171.57±84.24	29.90	0.73	18.62	0.48
PPG	174.63±87.69	26.84	0.36	20.07	0.51
Normal	201.47±91.03	Reference		Reference	
	**Unadjusted**			**Adjusted for age and axial length**	
	**Mean CVI (%)**	**Mean differences (%)**	**P values**	**Mean differences (%)**	**P values**
OAG	64.34±0.19	4.45	0.02	4.91	0.01
PPG	65.37±0.15	3.42	0.01	3.58	0.005
Normal	68.81±0.14	Reference		Reference	

The adjusted mean difference of CVI in OAG eyes was 4.91% (*p* = 0.01) lower than healthy eyes. The adjusted mean difference of CVI in PPG eyes was 3.58% lower than normal eyes (*p* = 0.005).

## Discussion

In the very early stages of glaucoma, the diagnosis is difficult because structural damage and functional losses may not be apparent. Since RGCs are concentrated at the macula [12], macular thickness or subfoveal choroidal vascularity can be valuable parameters for the detection of glaucoma. Therefore, we evaluated choroidal vascularity in the eyes of patients with OAG, PPG, and compared with healthy eyes.

Wolf et al. evaluated retinal blood flow in patients with POAG and healthy controls using scanning laser ophthalmoscopy [13]. Their results showed 11% reduction in the mean dye velocity within major retinal arteries. In a study evaluating choroidal vasculature using indocyanine green angiography in patients with glaucoma and normal subjects, the normal group showed the homogenous early choroidal filling pattern but the glaucoma group revealed a heterogeneous pattern and delayed peripapillary choroidal filling [14]. They proposed that the patients with glaucoma might experience reduced choroidal blood flow.

In much of the current literature, CT has been used as a surrogate to assess structural changes in the choroid. As the choroid can be influenced by many physiological variables, a marked disparity exists on the impact of glaucoma on CT [15,16]. The measurement of thickness is only partially representative of the choroid’s overall structural change. Therefore, measurement of CVI may help to explain the role of choroidal vasculature in the development and progression of glaucoma.

Recently, a normative database of subfoveal CVI, TCA, LA, and SA was established using 345 subjects [9]. The study showed that unlike CT, CVI was independent from systemic and ocular factors such as IOP, AL, or age. The average subfoveal CVI in healthy eyes was found to be 65.61 ± 2.33% [17]. In our study when we compared the factors that affect SFCT to those that affect CVI, our results showed SFCT was related with many physiological factors. In univariate analysis, AL and age were associated with SFCT, but not statistically significantly associated with CVI. After adjusting for age and AL in multivariate linear regression analysis, the CVI of both OAG and PPG eyes were significantly reduced compared to healthy eyes. CVI was not influenced by the physiological factors and SFCT showed greater variability compared to CVI. As a result, CVI may be a more stable index to use as compared to CT.

Our study also revealed that OAG and PPG eyes had smaller LA of choroidal vessels than healthy eyes since OAG and PPG eyes had 0.45±0.13㎟ and 0.47±0.11㎟ in contrast to 0.55±0.14㎟ of healthy eyes. Considering our results, glaucomatous eyes have smaller vascular lumen suggesting that choroidal ischemia may influence the pathogenesis of glaucoma or may be a result of glaucomatous damage. CVI was not influenced by physiologic factors, which suggests CVI to be a possibly more reliable tool than CT.

This study has some limitations. First, we studied a relatively small sample of patients with glaucoma. But the high repeatability of our measurements for the differences between the glaucomatous eyes and healthy eyes showed reliably. Second, the OCT measurements were not performed at the same time of the day. Previous study showed that macular choroidal volume had a circadian pattern with higher values at night and lower values during the day [18]. Since the OCT measurements were taken during the day time, the circadian changes were not likely to have affected our results. Third, topical IOP lowering medications may have influenced the vascular measurements. Previous work has shown that choroidal perfusion, detectable by an OCT-based system, is reduced in response to elevated IOP [19]. Further studies are needed to determine the impact on IOP lowering medications on OCT measurements.

Despite the above limitations, our study contributes to the understanding on the effect of glaucoma on the choroid. The use of CVI to illustrate phenotypical changes in the choroid in glaucomatous eyes is the major strength of this study. Other strengths include standardized data collection and imaging protocol.

In conclusion, we have shown that eyes with glaucoma have reduced choroidal vascularity as indicated by reduced CVI and that CVI may be a useful tool in studying vascular dysfunction in glaucoma. Our results support that vascular dysfunction and choroidal ischemia may be a contributory mechanism in the pathogenesis of glaucoma.
